# Three-dimensional architecture of podocytes revealed by block-face scanning electron microscopy

**DOI:** 10.1038/srep08993

**Published:** 2015-03-11

**Authors:** Koichiro Ichimura, Naoyuki Miyazaki, Shoji Sadayama, Kazuyoshi Murata, Masato Koike, Kei-ichiro Nakamura, Keisuke Ohta, Tatsuo Sakai

**Affiliations:** 1 Department of Anatomy and Life Structure, Juntendo University Graduate School of Medicine, Tokyo, Japan; 2 Institute of Physiological Sciences, Okazaki, Japan; 3 FEI Company Japan Ltd., NanoPort Japan, Tokyo, Japan; 4 Department of Cell Biology and Neuroscience, Juntendo University Graduate School of Medicine, Tokyo, Japan; 5 Laboratory of Morphology and Image Analysis, Center for Biomedical Research Resources, Juntendo University Graduate School of Medicine, Tokyo, Japan; 6 Division of Microscopic and Developmental Anatomy, Department of Anatomy, Kurume University School of Medicine, Kurume, Japan

## Abstract

Block-face imaging is a scanning electron microscopic technique which enables easier acquisition of serial ultrastructural images directly from the surface of resin-embedded biological samples with a similar quality to transmission electron micrographs. In the present study, we analyzed the three-dimensional architecture of podocytes using serial block-face imaging. It was previously believed that podocytes are divided into three kinds of subcellular compartment: cell body, primary process, and foot process, which are simply aligned in this order. When the reconstructed podocytes were viewed from their basal side, the foot processes were branched from a ridge-like prominence, which was formed on the basal surface of the primary process and was similar to the usual foot processes in structure. Moreover, from the cell body, the foot processes were also emerged via the ridge-like prominence, as found in the primary process. The ridge-like prominence anchored the cell body and primary process to the glomerular basement membrane, and connected the foot processes to the cell body and primary process. In conclusion, serial block-face imaging is a powerful tool for clear understanding the three-dimensional architecture of podocytes through its ability to reveal novel structures which were difficult to determine by conventional transmission and scanning electron microscopes alone.

Podocytes develop a characteristic architecture specialized for glomerular ultrafiltration, and are traditionally divided into three kinds of subcellular compartment; the cell body, primary process, and foot process^1^. The cell body of podocyte possesses several thick primary processes, each of which subsequently projects a number of fine foot processes (Fig. 1a, b). Adjacent podocytes are interdigitated with each other at their foot processes, which are separated from each other by filtration slits and bridged with a specialized intercellular junction, called a slit diaphragm (Fig. 1c). The foot processes and slit diaphragm serve as an adhesive apparatus to the glomerular basement membrane (GBM) and as a filtration barrier together with GBM and endothelial glycocalyx, respectively.

To explore the three-dimensional ultrastructure of podocytes in normal and pathologic conditions, conventional scanning electron microscopy (SEM) has been widely used^2^,^3^, since the luminal surface of podocytes faces the urinary space of Bowman's capsule and is easily observed (Fig. 1b, c). With conventional SEM, however, it is almost impossible to observe the basal surface of podocytes and some parts of podocytes situated within the deep vales, which are formed between the glomerular capillary loops. Thus conventional SEM would not be sufficient to examine the whole architecture of individual podocytes.

Reconstruction from serial sectional images is another technique to analyze three-dimensional structures of cell and tissue. Combination of transmission electron microscopy (TEM) of serial ultrathin sections and reconstruction from the serial images has been one of the most reliable methods for three-dimensional evaluation of external and internal cellular structures^4^,^5^,^6^,^7^,^8^. For example, in the nephrology field, the three dimensional configuration of subpodocyte space and primary cilia in immature podocytes were clearly demonstrated using serial ultrathin sections^9^,^10^. However, this technique is not widely used, because it extremely labor-intensive and time-consuming and requires considerable skill to manipulate large numbers of serial ultrathin sections.

Serial block-face imaging is a novel SEM technique, which enables much more efficient acquisition of a series of ultrastructural sectional images than the previous method. A block-face image can be obtained directly from a smooth surface of resin-embedded specimen using SEM, and the contrast-inverted block-face image is quite similar to the conventional TEM image of an ultrathin section^11^,^12^,^13^. Such SEM images can be serially and automatically acquired in a depth direction using a special SEM equipped with a system to remove a thin layer from the resin-embedded sample. At present, two types of SEM are commercially available for serial block-face imaging. One is a focused ion beam/scanning electron microscope (FIB/SEM) using focused beam of gallium ion for the removal^12^,^13^, and the other is a serial block face-scanning electron microscope (SBF-SEM) using an intrachamber diamond knife^11^. Recently three-dimensional reconstruction of subcellular structures based on serial block-face images has been widely applied in a variety of biomedical research fields, especially neuroscience. This method should also be useful for the structural analysis of renal tissues and cells including podocytes, but there has been no application of this method in this field, except for a recent study which clearly confirmed the glycocalyx layer of glomerular endothelial cells and the subpodocyte space^14^.

In the present study, we applied the three-dimensional reconstruction based on serial block-face images to analyze the whole architecture of podocytes from normal rat glomeruli, and also compared the reconstructed images generated from the serial images obtained from SBF-SEM and FIB/SEM. Our present results enabled to understand three-dimensional structure of podocytes more fully than ever before, and indicated that the serial block-face imaging is one of the powerful tool to examine the ultrastructural architecture of podocytes.

## Results

### Block-face images of podocytes

We obtained serial block-face images of podocytes from a perfusion-fixed rat kidney using SBF-SEM and FIB/SEM. Contrast-inverted block-face images achieved a quality comparable to conventional TEM images (Supplementary Fig. S1). However, several differences were found between conventional TEM and contrast-inverted block-face images (Fig. 2).

**Figure 1:**
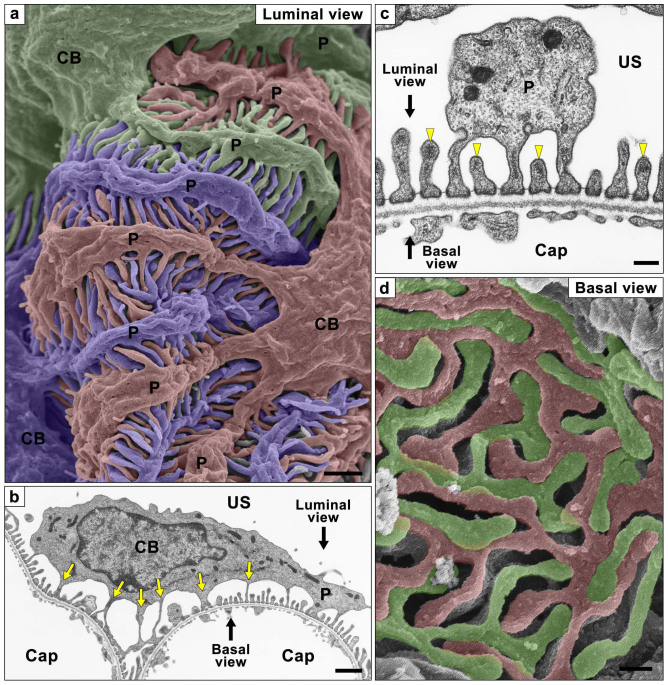
Podocyte subcellular compartments shown by conventional SEM and TEM. Podocyte are traditionally divided into three kinds of subcellular compartment, cell body (CB), primary process (P) and foot process. (a) Conventional SEM image shows the luminal surface of all three kinds of podocyte subcellular compartments. Three neighboring podocytes are individually colored with blue, green, and red. (b, c) Conventional TEM images also show the three subcellular compartments. The foot processes are predominantly protruding from the primary processes, but some of them are from the undersurface of cell body (yellow arrows in b). The foot processes possess the electron-dense actin bundle within their luminal cytoplasm (yellow arrowheads in c). Directions of luminal and basal views are indicated by black arrows. (d) Alkaline-maceration SEM images clearly show the basal surface of neighboring podocytes. The region in contact with the glomerular basement membrane consists almost entirely of the interdigitating foot processes. Two neighboring podocytes are individually colored with green and red. Cap, capillary lumen. US, urinary space of the Bowman's capsule. Bar scales, 1 μm in (a), (b); 200 nm in (c), (d).

**Figure 2:**
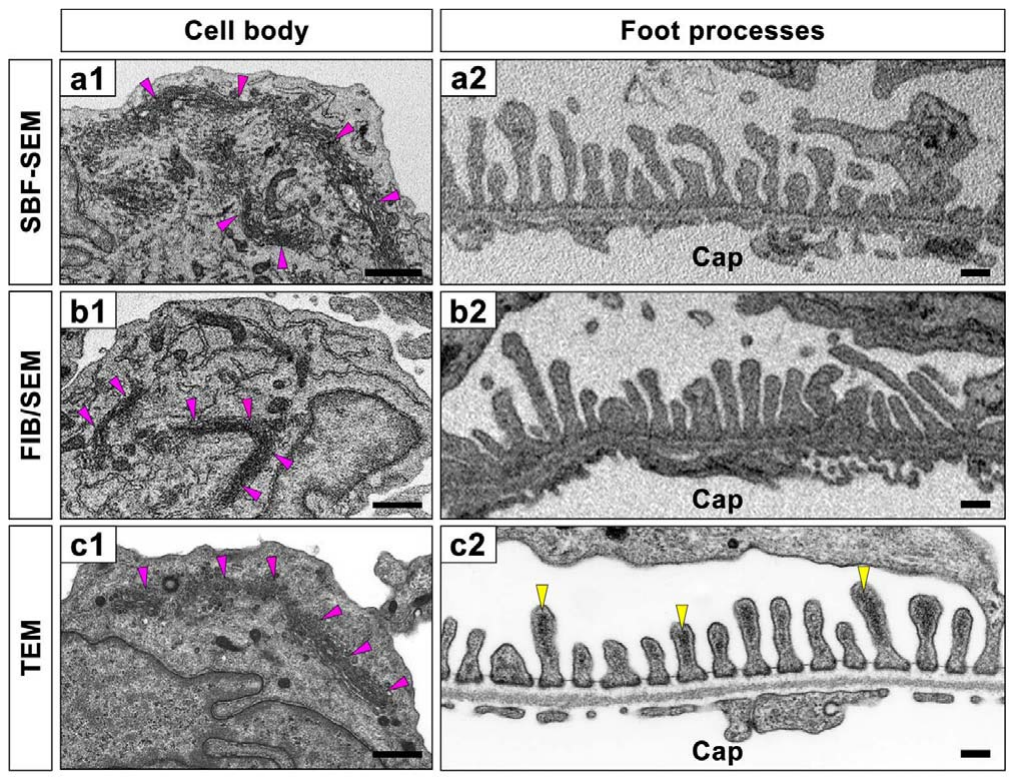
Comparison with block-face SEM and conventional TEM images of podocytes. The block-face images obtained with SBF-SEM (a1, a2) and FIB/SEM (b1, b2) are similar to the conventional TEM images (c1, c2). Due to the combinatorial heavy metal *en bloc* staining, the Golgi apparatus and endoplasmic reticulum in the cell bodies of podocytes are electron-densely depicted (pink arrowheads in a1, b1, c1). The electron-dense actin bundles, which are visualized at the luminal cytoplasm of the foot processes in the conventional TEM image (yellow arrowheads in c2), are difficult to see in the FIB/SEM and SBF-SEM images. Cap, capillary lumen. Bar scales, 500 nm in (a1), (b1), (c1); 200 nm in (a2), (b2), (c2).

The endoplasmic reticulum and Golgi apparatus were more clearly visualized in the block-face images (arrowheads in Fig. 2a1, b1) than in conventional TEM image (arrowheads in Fig. 2c1). This difference was attributed to the combinatorial heavy metal staining using reduced osmium tetroxide required for the block-face imaging. In general, postfixation with reduced osmium tetroxide has been known to be useful to visualize membrane organelles, such as the endoplasmic reticulum and Golgi apparatus^15^. However, block-face imaging was not suitable for the observation of podocyte cytoskeleton including actin cytoskeleton. Conventional TEM clearly depicted the prominent actin bundles in the foot processes as electron-dense materials, which were running along the longitudinal direction at the luminal half of the cytoplasm (arrowheads in Figs. 1c, 2c2)^16^,^17^. But, in the block-face images, the electron density appeared nearly homogeneous throughout entire cytoplasm of the foot processes, and the actin bundles were difficult to identify (Fig. 2a2, b2). This obscuration of actin bundles was presumably attributed to the maceration effect of osmium tetroxide against protein^18^. In the combinatorial heavy metal staining, specimens were immersed in osmium tetroxide solution at relatively high concentration and at room temperature, thus the maceration effect of osmium tetroxide was considered to be more pronounced.

### Three-dimensional reconstruction of podocytes from serial block-face images

**Figure 3:**
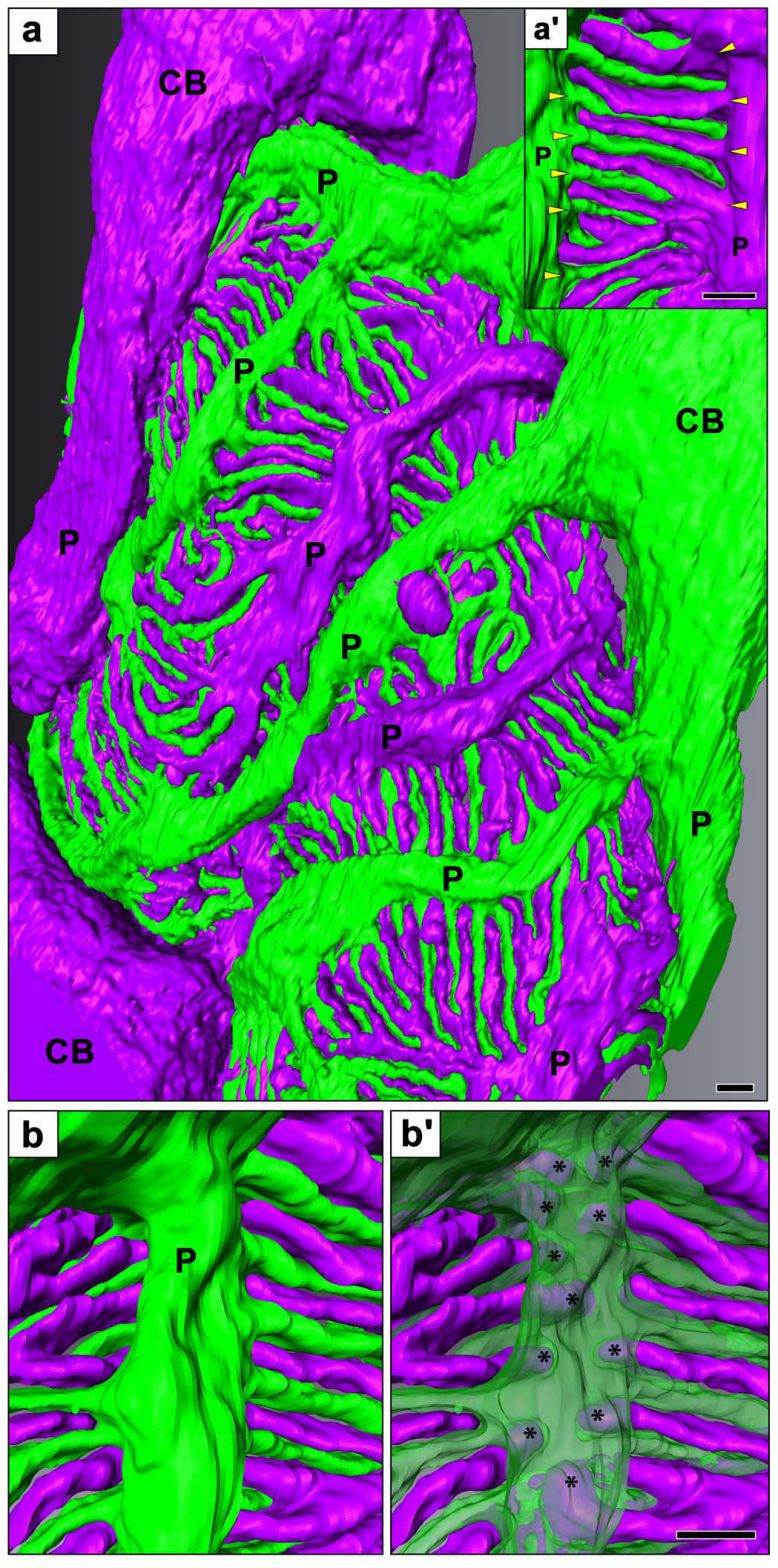
Luminal surface of reconstructed podocytes based on serial FIB/SEM images. Individual podocytes are represented with different colors. (a) The reconstructed image is quite similar to the conventional SEM images as shown in Figure 1a. (b, b′) These two reconstructed images are shown in the same region, and the green-colored podocyte is displayed semi-transparently in b′. The distal ends of foot processes are situated under the primary process (asterisks in b′), and both lateral surfaces of the ridge face the distal ends of foot processes. Bar scales, 1 μm in (a), 200 nm in (b′). These three-dimensionally reconstructed podocytes can be observed from any directions on the reconstruction software. CB, cell body of podocyte; P, primary process. (See also Supplementary Movies S1, S2).

**Figure 4:**
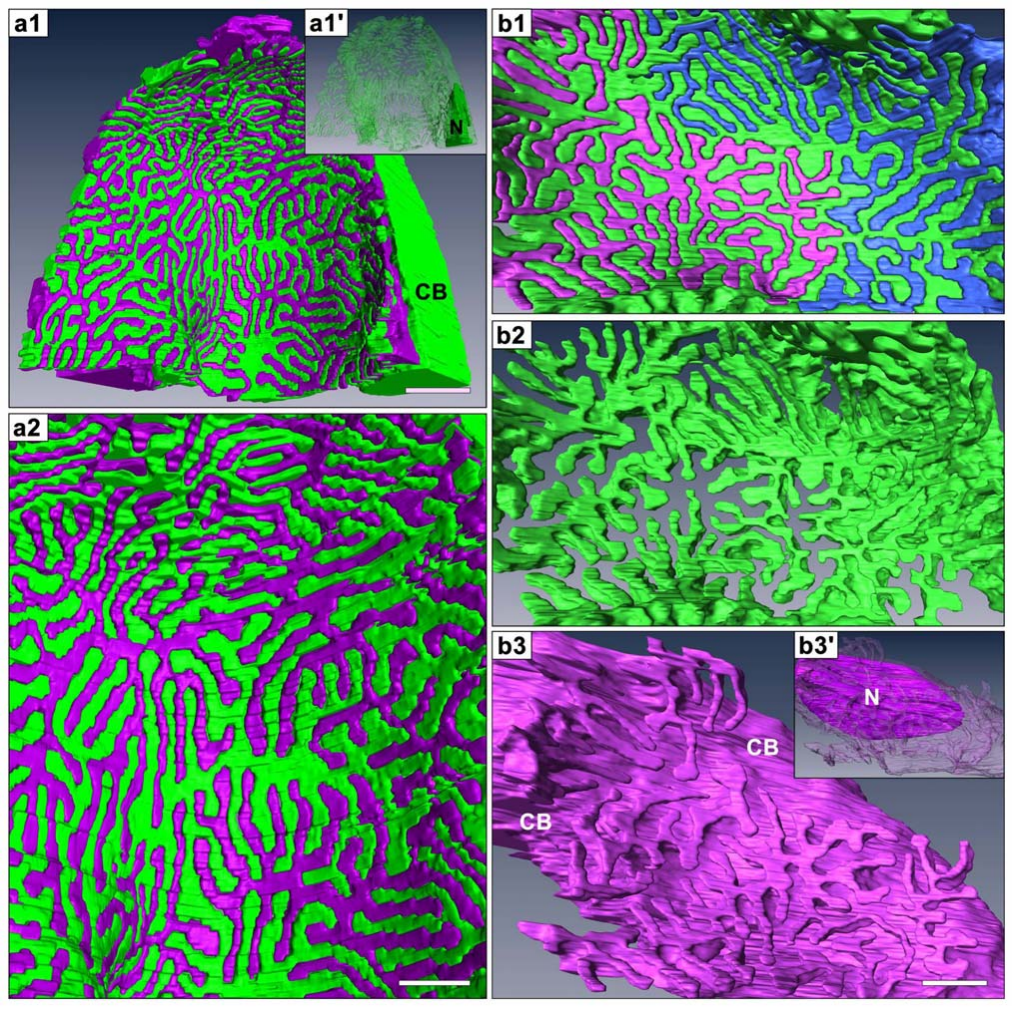
Basal surface of reconstructed podocytes based on serial SBF-SEM (a1, a2) and FIB/SEM (b1–b3) images. Individual podocytes are represented with different colors. (a1, a2, b1) These reconstructed images are quite similar to those of alkaline-maceration SEM as shown in Figure 1d, d′. The region where podocytes are in contact with the GBM consisted almost entirely of the interdigitated foot processes. (b2, b3) When the separate podocytes are individually observed from the basal side, the foot processes from the green-colored podocyte mainly protrude from the primary processes (b2), and the foot processes from the purple-colored one from the cell body (b3). The nucleus (N) in the cell body (CB) is visualized by clearing the surface and cytoplasm of cell body (a1′, b3′). Bar scales, 1 μm in (a1); 200 nm in (a2), (b3).

We segmented individual podocytes on serial block-face images obtained by FIB/SEM and SBF-SEM, and then reconstructed the segmented podocytes using an AMIRA 5.1 image analysis and reconstruction software. The three-dimensional reconstruction images based on the serial FIB/SEM and SBF-SEM images appeared quite similar to conventional SEM images (Figs. 3, 4, Supplementary Movie S1). However, the surface of podocytes appeared smoother in the FIB/SEM-based reconstruction than in the SBF-SEM-based one, because the electrical distortion of each block-face image due to surface charging was less in the FIB/SEM images.

The reconstructed podocytes could be rotated and seen from not only luminal side but also basal side, which would be one of the most important advantages of this method in the structural analysis of podocytes. When viewing the reconstructed podocytes from the luminal side, the foot processes appeared to be simply branched from the lateral aspects of the primary process, as the side branches from the axial major trunk in a plant leaf vein (Fig. 3a, a′), as shown by conventional SEM (Fig. 1a). However, the situation was certainly not that simple when viewed from the basal surface (or “the sole”) of the reconstructed neighboring podocytes. The contacting regions of podocytes to the GBM consisted almost entirely of interdigitating foot processes, which were approximately constant in width except for their slightly swollen ends (Fig. 4a1, a2, b1, Supplementary Movie S1).

To examine whether these basal surface images of the reconstructed podocytes exactly represented the real morphology, we also performed alkaline-maceration SEM, which enables direct observation of the basal surface of podocytes by removing the endothelial cells and extracellular matrices including GBM^19^,^20^. The basal surface images of the reconstructed podocytes were relatively consistent with alkaline-maceration SEM images (Figs. 1d, 4a2, 4b1), although the interdigitating foot processes were somewhat dissociated from each other due to the loss of supportive GBM in the alkaline-maceration SEM images (Fig. 1d).

**Figure 5:**
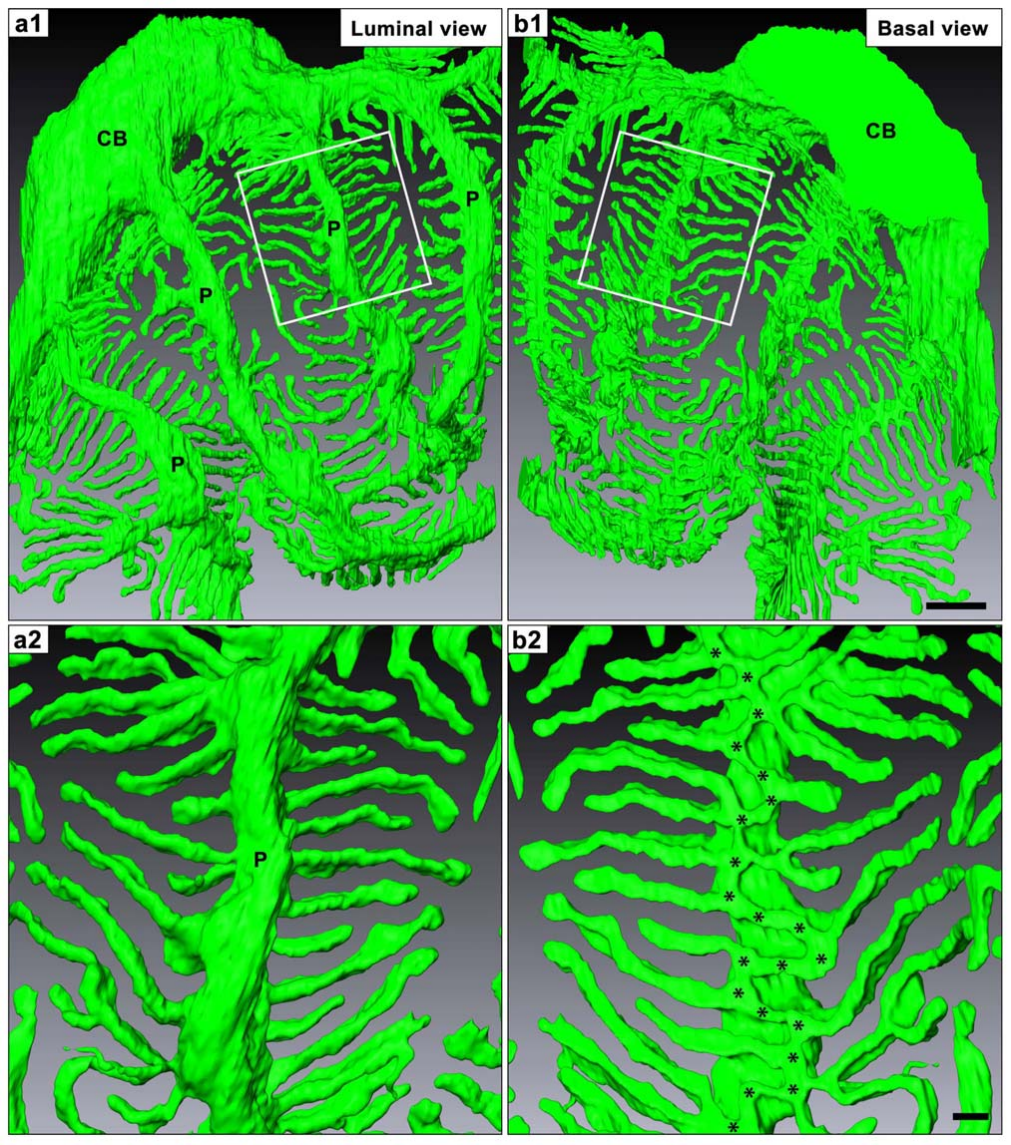
Three-dimensional structure of podocyte foot processes. A single reconstructed podocyte based on serial FIB/SEM images is observed from luminal (a1, a2) and basal (b1, b2) sides. (a2 and b2) are the magnification of the area indicated by rectangles in (a1 and b1), respectively. In the luminal surface view, the foot processes appeared to be simply branched from the lateral aspects of primary process, as found in a plant leaf vein (a2). In the basal surface view, the most proximal portions of foot processes are connected each other via a tortuous ridge-like prominence, which was formed on the basal surface of the primary process (asterisks in b2). The ridge-like prominences was quite difficult to predict their existence only by the conventional SEM observation from the luminal side, and was structurally similar to the usual foot processes. CB, cell body; P, primary process. Bar scales, 1 μm in (b1); 200 nm in (b2). The three-dimensionally reconstructed podocyte can be observed from any direction on the reconstruction software. (See also Supplementary Movies S1, S2).

**Figure 6:**
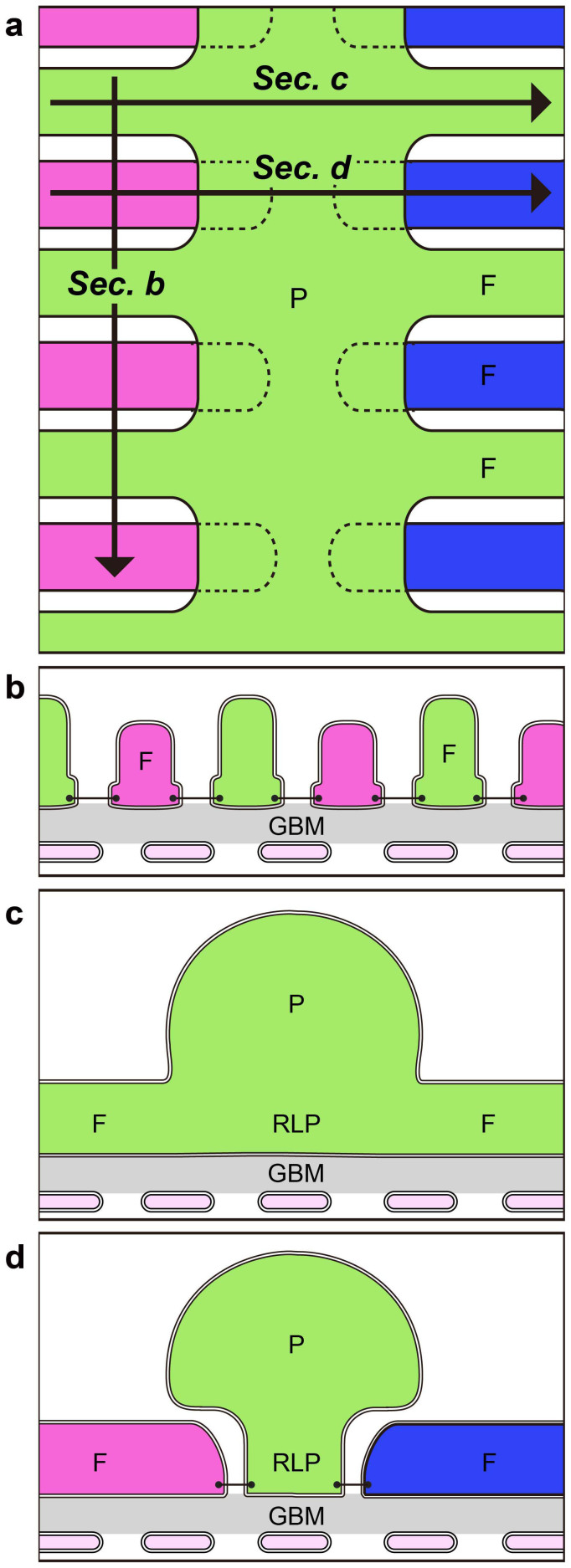
Structural relation of ridge-like prominence and foot processes. (a) Luminal surface view of podocytes shows primary and foot processes. Cross sections at the sites indicated by arrows (Sec. b, c, d) are shown in (b, c, d), respectively. (b) Podocytes adhere to the glomerular basement membrane with their foot processes. (c, d) The basal portion of primary process form the ridge-like prominence, and the primary process adheres to the glomerular basement membrane via the ridge-like prominence (RLP). The foot processes also protrude from the primary process via the ridge-like prominence. F, foot process; GBM, glomerular basement membrane; P, primary process.

To reveal the structural relationship among the three subcellular compartments of podocytes, we next observed single reconstructed podocyte which was separated from neighboring podocytes (Fig. 5, Movies S1, S2). When viewed from the basal side of the primary processes, we found that the foot processes were branched from a ridge-like prominence, which was protruded from the basal surface of the primary process (Fig. 5b1, 5b2) and was directly attached to the GBM like the foot processes (Supplementary Fig. S2). The distal ends of foot processes made their way under the primary process, and connected to the lateral surface of the ridge-like prominence via the slit diaphragm (Figs. 3b, 3b′, 6).

The foot processes were believed to be terminal projections protruded from the primary processes in general, but projections similar to foot processes were observed by TEM to be protruded from the cell body (arrows in Fig. 1b). When viewing a single reconstructed podocytes from the basal side, we could easily see that the foot processes also emerged from the cell body (Fig. 4b3, b3′). The way the foot processes protruded from the cell body was essentially similar to that found in the primary process. That is, the foot processes were connected with the cell body through the ridge-like prominence which directly protruded from the cell body. The ridge-like prominences from the cell body were often continuations of those from the primary processes at the transitional region between the cell body and primary processes.

## Discussion

**Figure 7:**
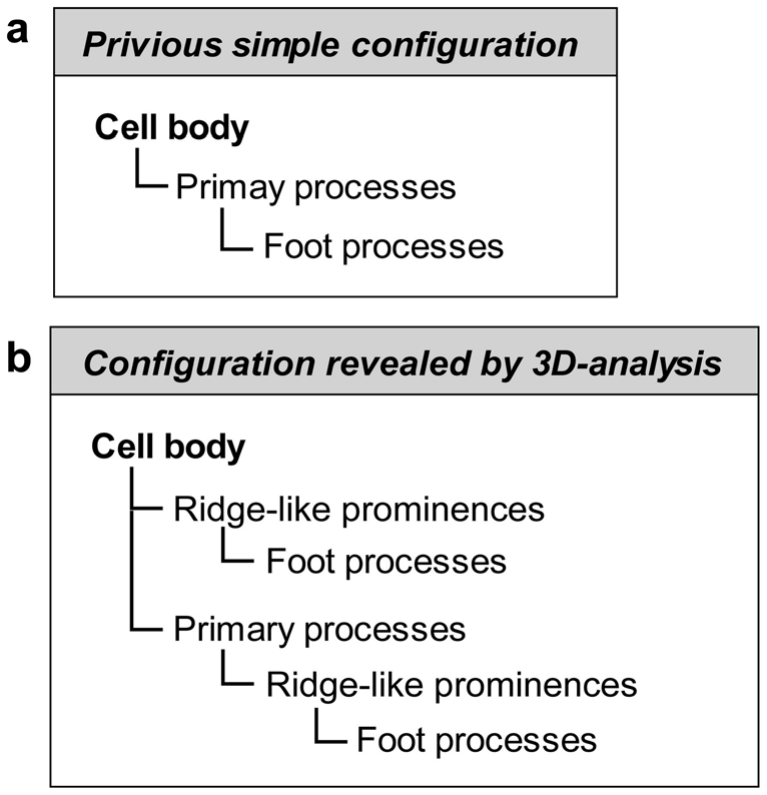
Structural hierarchy of podocyte subcellular compartments. (a) Previous simple configuration. The three subcellular compartments of podocyte are simply connected in this order. (b) Newly proposed configuration based on the present three-dimensional analysis. The ridge-like prominences, which are formed at the basal surface of the cell body and primary processes, serve as an adhesion apparatus for the attachment of cell body and primary processes to the glomerular basement membrane, and as a connecting apparatus of foot processes to cell body and primary processes.

It was previously believed that podocytes are divided into three kinds of subcellular compartment: cell body, primary process, and foot process, which are simply aligned in this order (Fig. 7a)^1^. From the present analysis of the three-dimensionally reconstructed podocytes, we proposed that a more accurate structural configuration of podocyte subcellular compartments would include ridge-like prominences (Figs. 6, 7b). The ridge-like prominences, which are protruded directly from the basal surface of the cell body and primary processes, serve as an adhesion apparatus for the direct attachment of cell body and primary processes to the GBM and as a connecting apparatus of foot processes to cell body and primary processes. The ridge-like prominences have been also described as “central foot processes” in the previous observation of a single isolated podocyte by alkaline-maceration SEM^20^.

In the present study, we examined only the mature podocytes in normal rats, but the serial block-face imaging and three-dimensional reconstruction should also be useful to analyze the structural maturation of podocytes during normal development and reorganization under pathologic conditions. In a variety of glomerular diseases, podocytes dynamically altered their external and internal structures. Some of these morphological alterations such as “foot process effacement” help to determine pathologic diagnosis in several glomerular diseases^21^,^22^. In this phenomenon, the normal interdigitated foot processes are finally reorganized into a broad flattened process like a paddle. The morphological process of the foot process effacement has not fully elucidated. The authors are now reevaluating these morphological processes using serial block-face imaging, and will report these data in the near future.

We also think that serial block-face imaging could be also a powerful tool to visualize and analyze intracellular structures including organelle and cytoskeletons in podocytes, as reported in other cell types^23^,^24^,^25^,^26^. Cytoskeletons are essential to form and maintain the unique architecture of podocytes^27^,^28^, and the three-dimensional information on the cytoskeletal arrangement is thus crucial to understanding the (re)organization of podocyte architecture in health, development, and disease. However, we found that the present protocol for the sample preparation for serial block-face imaging made it difficult to visualize actin cytoskeletons. The optimization of the protocol to visualize both cytoskeletons and organelles would be highly desirable. This problem may be addressed alternatively by pre-embedding immunogold labeling for specific proteins. The secondary antibody conjugated with 1.4-nm nanogold particles, which can be enlarged by sliver/gold enhancement technique, is used in this method. Enhanced nanogold particles can be easily visualized by serial block-face imaging, since their electron density is extremely high compared to those of other structures. Such immunogold serial block-face imaging has been already successfully applied to determine the specific type of synapse^29^. The foot processes were approximately constant in width except for their slightly swollen ends, suggesting that something at the end is different from the rest of the foot process (e.g. cytoskeleton, cell adhesion molecule). The combination of nanogold and enhancement techniques would be also helpful to reveal the molecular differences.

The basic architecture of podocytes is highly conserved throughout all of the vertebrate classes, but a variety of structural modification and specialization are found in each group^17^,^20^,^30^. The present results are based on a limited number of reconstructed podocytes from three rats. To comprehend the three-dimensional architecture in general, it is necessary to compare the three-dimensional architecture of podocyte among multiple vertebrate species including human using serial block-face imaging. Furthermore, certain cell types similar to the vertebrate podocytes both in structure and function have been reported in most invertebrate groups^31^,^32^,^33^. Three-dimensional architecture of such cell types has not elucidated so far in most of the invertebrate species. The block-face imaging should be useful to reveal the precise and accurate architecture of these cell types.

In conclusion, serial block-face imaging is a powerful tool for understanding the three-dimensional architecture of podocytes and revealing in more detail their structures which were difficult to determine by conventional TEM and SEM alone. Furthermore, on the basis of this analysis, we could propose a more accurate structural configuration in podocytes.

## Methods

### Fixation of animals

Three male Wistar rats (8 weeks) were obtained from Charles River Laboratories Japan (Yokohama, Japan). Animals were perfused with physiological saline and subsequently 2.5% glutaraldehyde fixative buffered with 0.1 M phosphate buffer under anesthesia with pentobarbital. All the procedures performed on laboratory animals were approved by the Institutional Animal Care and Use Committee of Juntendo University School of Medicine (approval No.250042). All the animal experiments were carried out in accordance with the Guidelines for Animal Experimentation of Juntendo University School of Medicine.

### Conventional and alkaline-maceration SEM

Small cubes of fixed kidney cortex were immersed in 2% osmium tetroxide in 0.1 M phosphate buffer for 2 hr. After dehydration with graded series of ethanol, specimens were transferred to *t*-butyl alcohol, and freeze-dried with an ES-2030 freeze dryer (Hitachi High-Technologies, Tokyo, Japan). After mounting on aluminum stubs with carbon paste, the dried specimens were coated with osmium with an OPC80T osmium plasma coater (Filgen, Nagoya, Japan), and observed with an S-4800 scanning electron microscope (Hitachi High-Technologies). Some fixed specimens were processed by alkaline maceration according to the previous method with some modifications^19^,^34^,^35^. Briefly, the small cubes of fixed kidney cortex were placed in 6 M sodium hydrate for five min at 60°C, and then for 10 min at 45°C. After alkaline maceration, the tissues were rinsed in 0.01 M phosphate buffer (pH 7.3) containing 0.05% Tween 20 overnight. The macerated tissues were transferred into 50% aqueous solution of dimethyl sulfoxide, and then freeze-cracked in liquid nitrogen. The cracked samples were processed as were in conventional SEM protocol as described above.

### Sample preparation for FIB/SEM and SBF-SEM

The fixed kidney samples were cut into 250-μm-thick slices with a DTK-1000 Microslicer (Dosaka EM, Kyoto, Japan), and the slices were processed largely in accordance with a combinatorial heavy metal staining protocol which has been released on the website of the National Center for Microscopy and Imaging Research (La Jolla, CA) (http://ncmir.ucsd.edu/sbfsem-protocol.pdf). This protocol was designed to enhance signal for backscatter electron imaging of epoxy-resin-embedded mammalian tissue at low accelerating voltages. In brief, the tissue slices were successively immersed in 1% osmium tetroxide containing 1.5% potassium ferrocyanide in 0.1 M cacodylate buffer for 1 hr on ice, 1% low molecular weight tannic acid (Electron Microscopy Sciences, Hatfield, PA) in 0.1 M cacodylate buffer for 4 hr at RT, 2% aqueous osmium tetroxide for 30 min at RT, 1% aqueous uranyl acetate overnight at RT, and Walton's lead aspartate solution^36^ for 30 min at 60°C. The slices were then dehydrated with a graded series of ethanol, and were embedded in epoxy resin, Oken Epok 812 (Okenshoji, Tokyo, Japan).

### FIB/SEM

The surface of resin-embedded tissue was exposed using a diamond knife on an Ultracut UCT (Leica Microsystems, Vienna, Austria). The blocks were mounted onto an aluminum stub, and then coated with a thin layer of heavy metal to prevent charging. The surface of embedded tissue was imaged with a Helios Nanolab 650 FIB/SEM (FEI, Eindhoven, Netherlands) at a high acceleration voltage of 20 kV to find the area of interest. New surface for serial block-face imaging was generated by FIB-milling at a 2.5 nA beam current, where gallium ions were accelerated by a voltage of 30 kV. Serial block-face images were obtained every 50-nm depth with a backscattered electron detector at an acceleration voltage of 1.9 kV. The pixel size was 16.5 nm/pixel wide, 21.0 nm/pixel height, 50 nm/pixel depth, and the pixel dimensions of a recorded image were 3072 × 2048 pixels. The contrast of the images was inversed.

### SBF-SEM

Small pieces of block including glomerulus were trimmed and mounted on aluminum specimen pins (Gatan, Pleasanton, CA) using CircuitWorks Conductive Epoxy (Chemtronics, Kennesaw, GA). The entire surface of the specimen was coated with a thin layer of heavy metal. New surface for serial block-face imaging was generated using a 3View in-chamber ultramicrotome (Gatan) within a ΣIGMA/VP SEM (Carl Zeiss Microscopy, Jena, Germany). Block-face images were obtained every 70-nm depth with a backscattered electron detector at an acceleration voltage of 1.1 kV. The pixel size was 21.0 nm/pixel wide, 21.0 nm/pixel height, 70 nm/pixel depth, and the pixel dimensions of a recorded image were 4096 × 4096 pixels. The contrast of the images was inversed.

### Data processing for three-dimensional reconstruction

For alignment of serial SBF-SEM images, a Fiji open source software (http://fiji.sc/Fiji) was used. Segmentation and three-dimensional reconstruction of podocytes were performed using an AMIRA 5.1 Software (FEI Visualization Science Group, Burlington, MA) for both FIB/SEM and SBF-SEM data. The segmentation of each podocytes was easily performed, since the serial block-face images of glomerular wall were similar to conventional TEM images and exhibited enough high contrast to identify the three kinds of glomerular cells, mesangial matrix, and GBM. The three-dimensional reconstruction images of podocytes from serial block-face images using SBF-SEM and FIB/SEM showed highly consistent results with the conventional and alkaline-maceration SEM images, indicating that the segmentation of individual podocytes from each block-face image and reconstruction of segmented podocytes were adequately performed.

## Author Contributions

K.I. designed the experiments. K.I., N.M., S.S., K.M., K.N. and K.O. performed experiments. K.I., M.K. and T.S. analyzed the experimental data. K.I. prepared all figures and wrote the main manuscript text. All authors reviewed the manuscript.

## Supplementary Material

Supplementary Information Supplementary information

Supplementary Information Supplementary Movie S1

Supplementary Information Supplementary Movie S2
