# Supplementary material for: Three-dimensional architecture of podocytes revealed by block-face scanning electron microscopy
Source: Sci Rep. 2015 Mar 11;5:8993. doi: 10.1038/srep08993 (PMC4355681; doi:10.1038/srep08993)
Supplement: Supplementary Information [file srep08993-s1.pdf]

Supplementary information

Three-dimensional architecture of podocytes revealed by block-face scanning electron microscopy

Koichiro Ichimura, Naoyuki Miyazaki, Shoji Sadayama, Kazuyoshi Murata, Masato Koike, Kei-ichiro Nakamura, Keisuke Ohta, and Tatsuo Sakai

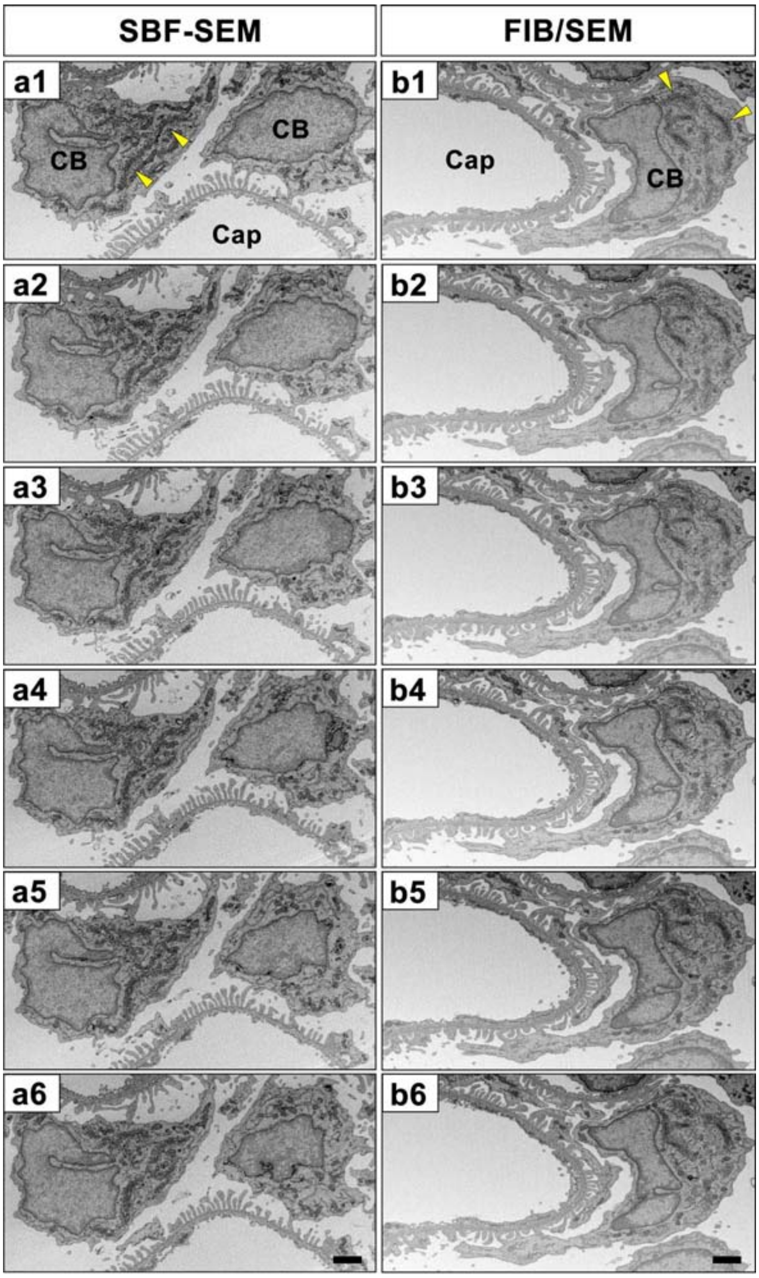

Supplementary Figure S1. Serial block-face images of glomerular wall obtained with SBF-SEM (**a1-a6**) and FIB/SEM (**b1-b6**). The block-face images are quite similar to the conventional TEM images. Due to the combinatorial heavy metal *en bloc* staining, the Golgi apparatus and endoplasmic reticulum in the cell bodies of podocytes are electron-densely depicted (arrowheads in **a1, b1**). CB, cell body of podocyte; Cap, capillary lumen. Bar scales, 500 nm.

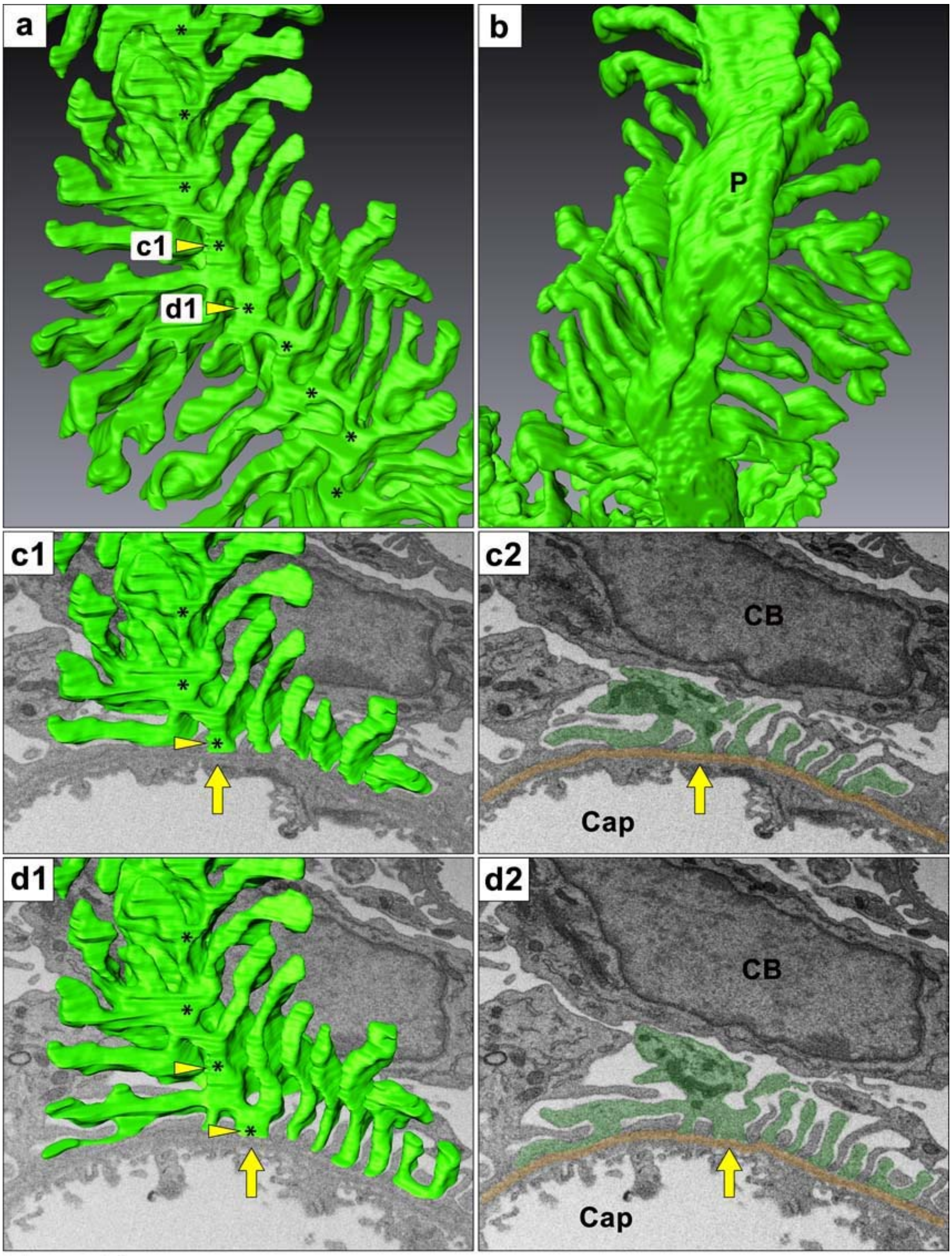

Supplementary Figure S2. Structural relation of ridge-like prominence and glomerular basement membrane. Three-dimensional reconstruction of one primary process and foot processes originating from it is shown from basal (**a**) and luminal (**b**) sides. The ridge-like prominence is indicated by asterisks. (**c1**-**2**, **d1**-**2**) Block-face images showing transverse section of the ridge-like prominence (arrows). As well as the foot processes, the ridge-like prominence directly adheres to the glomerular basement membrane (colored with blown in **c2**, **d2**). Cap, capillary lumen; CB, cell body of podocyte; P, primary process. Bar scales, 200 nm.

Supplementary Movie S1. Animation of the three-dimensionally reconstructed podocytes. This reconstructed image is obtained from the segmented volume of the serial FIB/SEM images, and corresponds to Figs 3 and 5. Three neighboring podocytes are contained in this reconstruction. To clearly depict the extension of a single podocyte, one podocyte is colored with green, and the other two with purple.

Supplementary Movie S2. Animation of the three-dimensionally reconstructed podocytes. To display the structural relationship between the connecting and peripheral foot processes in detail, we cropped and magnified one region of the reconstructed podocytes shown in Supplementary Movie S1.
